# The Criterion Validity, Reliability, and Feasibility of an Instrument for Assessing the Nursing Intensity in Perioperative Settings

**DOI:** 10.1155/2017/1048052

**Published:** 2017-07-17

**Authors:** Satu Rauta, Sanna Salanterä, Tero Vahlberg, Kristiina Junttila

**Affiliations:** ^1^Helsinki University Central Hospital, P.O. Box 340, 00029 HUS, Finland; ^2^Department of Nursing Science and Turku University Hospital, Turku University, 20004 Turku, Finland; ^3^Department of Biostatistics, Turku University, 20004 Turku, Finland; ^4^Helsinki University Central Hospital, P.O. Box 280, 00029 HUS, Finland

## Abstract

Patient classification systems generate information for staff allocation based on a patient's care needs. This study aims to test further the instrument for assessing nursing intensity (NI) in perioperative settings. Nine operating departments from five university hospitals were involved. The perioperative nurses gathered data from patients (*N* = 876) representing different fields of surgery. Reliability was tested by parallel classifications (*n* = 144). Also, the users' (*n* = 40) opinions were surveyed. The results support the predictive validity and interrater reliability of the instrument. The nurses considered the instrument feasible to use. The patients' low ASA class did not automatically signify low NI; however, high ASA class was more frequently associated with high intraoperative NI. Intraoperative NI indicated the length of the postanaesthesia care and the type of the follow-up unit. Parallel classifications ensured the homogenous use of the instrument. The use of the instrument is recommended.

## 1. Introduction

When scrutinizing the surgical patient's progression through the operation process from the referral to the follow-up appointment, the time spent in the operating department is the most expensive part of the care provided. Staff expenses comprise the majority of the total costs (Peltokorpi and Kujala [1]). Hence, it is of high importance to optimally allocate perioperative nursing staff resources. Decisions in nursing management should be based on knowledge, and the quality of the decision depends on the reliability of the available information. Therefore, reliable sources of information are essential. Patient classification systems that generate information about nursing intensity (NI) provide data that can be used for decisions concerning the optimal allocation of nursing staff resources.

Nurse staffing has mainly been based on tradition, rather than the NI, in any nursing context (Fagerholm [2]). There is evidence to indicate that adequate staffing levels and balanced nursing workloads reduce the intention to leave the profession (Flinkman et al. [3]), reduce sickness absenteeism (Rauhala et al. [4]), and increase job satisfaction among nurses (Cummings et al. [5], Jourdain and Chênevert [6], Kalisch and Lee [7], and Pineau Stam et al. [8]). Adequate staffing levels and balanced nursing workloads also produce better patient outcomes (Junttila et al. [9]).

However, new metrics are needed for successful workforce planning and staff allocation so that the care needs of our patients and the quality of care are ensured. A growing body of scientific research has been published concerning NI instrument called OPCq which is validated for use in the inpatient hospital wards (Fagerström and Rauhala [10], Fagerström [11], and van Oostveen et al. [12]). Similar instrument with scientific evidence for perioperative settings is not available. Edel has presented a perioperative patient acuity system as early as 1995. Association of periOperative Registered Nurses (AORN) has discussed using OR (=operating room) Patient Classification for Staffing Assignments by calculating together ASA Physical Status Classification and the complexity of surgical procedure [13].

In this study, we further tested the instrument designed for assessing NI in perioperative settings. Our interest was to investigate the correlation between the NI and the ASA Physical Status Classification System of the American Society of Anaesthesiologists (American Society of Anaesthesiologists [14]). The ASA Physical Status Classification is a simple method used by anaesthesiologists to estimate patients' preoperative physiological status and thus to assess the surgical patient's condition (Table 1). Compared to ASA Physical Status Classification, the instrument tested in this study is used by registered nurses to evaluate not the preoperative status but the patient's care needs during the whole nursing process (pre-, intra-, and postoperative phase of surgical patients' care).

The correlation between ASA Physical Status Classification and postoperative outcomes, postoperative complications, and mortality has been verified in several studies (Wolters et al. [15], Prause et al. [16], and Leung and Dzankic [17]). However, the correlation between ASA category and length of hospital stay has been supported by some studies (El-Haddawi et al. [18], Carey et al. [19], and Torkki [20]) but not others (Ranta et al. [21], Cuvillon et al. [22], and Daabiss [23]). Thus, we sought to study whether the evaluation of NI based on patient's care needs could add value to the ASA Physical Status Classification based on preoperative condition. Per se it can be presumed that the sicker the patients are preoperatively, the more care they might need. The ASA Physical Status Classification was chosen as a comparator because it is widely used both internationally and nationally.

Furthermore, we were interested in correlating NI and patient flow through the operating department. The flow was examined based on different time labels, which are routinely gathered from different phases of the surgical patient's transition through the operating department. By the patient flow we mean how smoothly the patient is moving through operating department: from operating room to Postanaesthesia Care Unit (PACU) and from PACU to ward.

The number of operations, operating time utilization, and turnover times are the most measured metrics in operating departments in Finland (Marjamaa and Kirvelä [24]), and these variables are also of common interest internationally (see, e.g., Iyer et al. [25] and Foley and Soldani [26]). Different time labels have been estimated to illuminate the patient flow through the perioperative continuum (Torkki et al. [27], Torkki [20]), and efforts have been made to reduce throughput times by minimizing the nonoperative time (Sandberg et al. [28], Krupka and Sandberg [29]). However, the results from these metrics never or seldom lead to any changes in staff allocation or recruitment (Marjamaa and Kirvelä [24]). We explored the predictive validity of the instrument and tested if the intraoperative NI could predict the length of stay in the Postanaesthesia Care Unit (PACU) and the need for follow-up care.

Third, we tested the reliability of the instrument through parallel classifications. This is a viable method in situations in which the target is changing and the assessments cannot be repeated (Grove et al. [30]).

In addition, it is of high importance that the user's opinion is heard during the development process of an instrument. The Oxford Dictionary (Oxford English Dictionary [31]) defines feasibility as “capable of being done, accomplished or carried out; possible; practicable when applied to a design or a project/capable of being dealt with successfully in any way, either in a material or immaterial sense when applied to things in general.” In this study, the feasibility was evaluated through concreteness, usability, understandability, clarity, and objectivity.

Perioperative nursing was defined as nursing performed by registered nurses who work at an operating department or day surgery unit as anaesthetic, circulating, scrub, or postanaesthesia care nurses (Junttila [32]). NI comprises both the direct and indirect patient care activities but not the non-patient care activities related to nurses' work (Morris et al. [33]). The scope of NI is broader than just the calculation of nurse-patient ratios (Welton et al. [34]) or the calculation of the amount of time spent in direct or indirect nursing care (Cusack et al. [35]). In perioperative settings nurse-patient ratios have been presented by ASPAN [36] for pre- and postoperative phases of care, and AORN [37] has determined the number of personnel per operating room in its Position Statement on perioperative safe staffing. On the other hand, in cases of difficult surgical procedures or multiple repetitive, fast surgical procedures, the need for an additional circulating nurse has been recognized by perioperative nurses themselves (Bell [13]).

However, Butler et al. [38] have stated that it is challenging to translate the traditional means of planning nurse staffing to perioperative settings. On the other hand, Upenieks et al. [39] argued that the major weakness of the nurse-patient ratio is that it ignores other factors that may significantly affect the nurses' workload, including patients' care needs. Generally, measurements which do not incorporate patients' acuity lack an important perspective because patients' need for nursing care is not reflected (Mark and Harless [40]). The starting point in evaluating NI is the patient's care needs and the nurse's response to these needs (Fagerström et al. [41], Rauhala and Fagerström [42], Andersen et al. [43], and Fagerström et al. [44]).

In conclusion, several arguments supported testing a new instrument. Valid instruments measuring surgical patients' care needs are not available for perioperative nursing care. ASA Physical Status Classification tends to be used by anaesthesiologists and the target is different. Furthermore, metrics such as nurse-patient ratios may not be applicable for operating departments' staffing purposes as a sole perspective.

## 2. Purpose of the Study

The purpose of this study was to test the validity, reliability, and feasibility of the NI instrument. The ultimate goal is to create a solid and user-friendly tool for assessing NI in different types of perioperative settings.

## 3. Methods and Material

### 3.1. Study Design

This study used an analytic, nonexperimental, cross-sectional design (Pai and Filion [45]).

### 3.2. The Study Instrument

The instrument for assessing NI in the perioperative setting was initially developed by a national workgroup and later named as PERIHOIq. Previously, the content validity of the instrument was shown to be acceptable (Rauta et al. [46]). Furthermore, the construct of the instrument was examined by principal component analysis. The model consisting of four principal components was suggested. Based on the model, the instrument for the operating room differed from the instrument for the PACU (Rauta et al. [47]). However, we chose to further evaluate one uniform instrument, accepting the fact that some nursing interventions are emphasized differentially in operating rooms and PACUs.

The instrument used in this study consisted of six categories and exhibited better clinical relevance than the four-category instrument. Furthermore, the six-category instrument followed a structure of patient classification that is in common use in other Finnish nursing contexts (e.g., wards, outpatient units). Categories were divided into subcategories, and three to five subcategories were included in each category. These subcategories described the core elements of perioperative nursing. The six main categories were as follows: (1) the planning and organization of the perioperative care, (2) physiological balance, (3) medication, pain, and nausea, (4) aseptic practice, (5) activity/rest, mobilization, and positioning, and (6) support, guidance, and continuity of care including specimens and examinations.

The NI was assessed within each category with a four-point scale from slight NI (=1) to demanding NI (=4) according to the maximum level of actualized NI. Finally, the total NI points were calculated by summing up the NI points of the six categories. The points could range from a minimum of 6 points to a maximum of 24 points. For reliability testing, five NI classes were included (Frilund and Fagerström [48], Andersen et al. [43], and van Oostveen et al. [12]).

### 3.3. Sample and Setting

Nine operating departments from five university hospitals in Finland were recruited to participate in this study. These departments comprised different fields of surgery from ambulatory to emergency surgery.

Data were collected from one to two months' period in stages from October 2008 to June 2009. Before the data collection period, 130 perioperative nurses were trained to use the instrument. The training consisted of the introduction of the instrument and the principles of how to use it. The use of the instrument was rehearsed by evaluating different patient cases representing surgical patients cared for in those units where the nurses were from. The introduction and principles sessions lasted four hours together and the case training sessions 3 hours per one case, altogether 9 hours. The sessions were divided between two days.

Trained nurses gathered the data from patients they cared for daily as part of the normal scheduled performance in their units. After the study period, these same nurses were surveyed to report the feasibility of the instrument.

The reliability was tested through parallel classifications. These data were collected separately during two-week periods in the same departments.

### 3.4. Data Collection

The nurse or the team of nurses involved in patient care classified each patient during their shift according to the guidelines on a paper form designed by the researchers. Altogether, 876 patients were involved. The total number of patients in each classification included 265 in the preoperative, 846 in the intraoperative, and 609 in the postoperative phase of care. These classifications were obtained from paediatric and adult patients undergoing ear, nose, and throat surgery, eye surgery, gynaecologic surgery, urologic surgery, plastic surgery, endoprosthesis surgery, and heart surgery. The types of surgery included day surgery, short stay surgery, and emergency surgery.

To test the interrater reliability, two nurses separately and independently evaluated the same patient. In all, 144 parallel classifications were obtained. Because the number of parallel classifications was only 144, we decided to analyze them as a whole. The number of parallel classifications per department varied from 16 to 27. However, this study was an instrument testing, not the testing of consensus between the classifications at department level, and thus we consider the amount of data acceptable.

Finally, after the testing period, those perioperative nurses who had used the instrument (*n* = 130) received a self-tailored electronic questionnaire to assess the feasibility of the instrument from the user's point of view. They were asked how well the instrument evaluated their patients' NI in general and by each main category separately. In response to these questions they answered on a four-point scale (1 = not well at all, 2 = not very well, 3 = quite well, and 4 = very well).

Also, the instrument's abilities to cover patients' care needs and on the other hand to separate these care needs from each other were asked about with the aforementioned scale. The respondents rated the concreteness, usability, understandability, clarity, and objectivity of the instrument.

In addition, the questionnaire included questions exploring the respondents' demographic details. The response rate for this survey was 31% (*n* = 40).

### 3.5. Data Management and Analysis

Statistical analyses were performed using SAS System for Windows, version 9.4 (SAS Institute Inc., Cary, NC, USA). The frequencies and percentages were calculated. Correlations between the total NI points and the ASA categories were examined with Spearman correlation coefficients. The differences in NI points between ASA categories in pre-, intra-, and postoperative phases were examined with the Kruskal-Wallis test. Additional pairwise comparisons were performed with the Mann–Whitney *U* test using Bonferroni-corrected *p* values. *p* values less than 0.05 were considered statistically significant.

The predictive validity was tested using Spearman correlation coefficients between the NI points and the time that the patients spent in the PACU. The differences in NI points between places of discharge from the PACU were tested with the Kruskal-Wallis test and pairwise comparisons with the Bonferroni-corrected Mann–Whitney *U* test. *p* values less than 0.05 were considered statistically significant.

The interrater reliability was tested using the agreement percentage. Two nurses performed independent classifications on the same patient (*n* = 144), and, all together, five NI classes were formulated based on singular NI points from both classifications. These classes were compared, and the agreement percentage was calculated. An agreement percentage of over 70% has previously been accepted as satisfactory (Frilund and Fagerström [48], Andersen et al. [43], and van Oostveen et al. [12]).

The data from the users' survey were analyzed by calculating percentages when appropriate. A content validity index was calculated concerning the qualities of the instrument (CVI, value by default 0-1) using a computer spreadsheet (Polit and Beck [49]). The number of scores of 3 and 4 was added and divided by the number of respondents (Lynn [50]). CVI values of at least 0.78 were considered to indicate good content validity (Polit et al. [51]).

### 3.6. Ethical Considerations 

The Finnish national legislation and ethical principles were taken into account when conducting this study. The five university hospital organizations that were involved in this study granted permission to conduct this study in accordance with their guidelines concerning scientific research.

The patients were not separately enrolled in the study, as their NI was evaluated as part of normal daily care. The participating nurses received written information about the study in connection with the data collection instrument and the voluntary nature of the study, and the right to refuse or withdraw from the study was explained to them. The nurses were voluntarily enrolled in the study, and their informed consent was regarded to be received when they returned the data collection forms.

The data were handled with confidentiality. No names or identity codes were collected from the patients at any time. The names of the attending nurses were collected only to send them the electronic survey afterwards, but, in their answers, their names were not displayed, so their anonymity was ensured.

## 4. Results

The highest number of patients, 846, was classified as being in the intraoperative phase of patient care, the second highest number of patients (609) was in the postoperative phase, and the lowest number of patients (265) was in the preoperative phase.

The highest median NI point value, 10, was assigned to the intraoperative phase, and the lowest median NI point value, 7, was assigned to the preoperative phase. In the postoperative phase, the median NI point value was 8. The minimum scores were 6 in all three phases, but the maximum number of NI points (24) was only assigned to patients in the intraoperative phase. In the postoperative phase, the maximum scores were higher (20) than in the preoperative phase (16).

There was a moderate positive correlation between NI points and ASA category in the intraoperative phase (*r* = 0.39, *p* < 0.0001). In the pre- and postoperative phases, the correlations were weak (*r* = 0.24, *p* < 0.0001 and *r* = 0.18, *p* < 0.0001, resp.).

There were statistically significant differences in NI points between different ASA categories in the pre-, intra-, and postoperative phases (Table 2). In the preoperative phase, ASA category IV patients showed additional NI points compared to patients in ASA category I or II (*p* < 0.001). There were also statistically significant differences when comparing ASA category III patients with ASA category I or ASA category IV patients (*p* < 0.05).

In the intraoperative phase, patients in ASA categories III and IV required high NI during the surgical procedure: patients in these categories had higher NI points compared to patients in ASA category I or II (*p* < 0.0001). In addition, patients with ASA category IV had higher points compared to patients in ASA category III (*p* < 0.0001).

In the postoperative phase, the findings were in line with other phases. The differences were not statistically significant when comparing ASA category I patients with patients in category II or III to IV.

The comparison of intraoperative NI with the PACU time revealed that the intraoperative NI correlated weakly with PACU time (*r* = 0.21; *p* < 0.0001). Thus, high NI during the surgical procedure indicated that the patient may require longer postoperative monitoring before being transferred to the follow-up unit. The PACU time indicated the amount of time that the patient was under persistent monitoring and surveillance in the PACU. The average PACU time was 2.4 hours, and the standard deviation was 1 hour and 15 minutes with a minimum of 15 minutes and a maximum of 13.3 hours. The intraoperative NI positively correlated with the duration of the surgical procedure (*r* = 0.49; *p* < 0.0001) and the time that the patients spent in the operating room (*r* = 0.55; *p* < 0.0001). Specifically, greater NI was associated with a longer surgical procedure and longer stay in the operating room.

Altogether, 607 classifications were involved in the analysis in which NI points were compared in the follow-up units. The follow-up unit options included the surgical ward, the intensive care unit (ICU), or home. According to the results, the preoperative, intraoperative, and postoperative NI predicted the patient's need for intensive care after the surgical procedure (*p* < 0.05) (Table 3). The patients from the day surgery units went home after recovering in the PACU, and they exhibited significantly lower intraoperative NI points than those patients who were transferred to the surgical wards or to the ICU (*p* < 0.0001).

### 4.1. Reliability

The parallel classifications, independently performed by two nurses, indicated satisfactory interrater reliability. The agreement percentage was over the limit value of 70% in all phases of surgical patient care. The agreement percentage was highest in the preoperative phase, 100%, and lowest in the intraoperative phase, 72%. In the postoperative phase, the agreement percentage was 78%.

### 4.2. Feasibility of the Instrument

The survey was sent to 130 perioperative nurses, and 40 responses were received; thus, the response rate was 31%. The respondents were experienced nurses; almost half of them had over ten years of work experience in perioperative nursing. In addition, these nurses worked equally in different roles in their departments. Only elective patients were operated on in one-fourth of the departments. Approximately 70% of respondents had previous experience using some type of patient classification system, so they had some previous knowledge of the instrument used in the assessment of NI.

The instrument was regarded to evaluate the patients' NI as a whole relatively well or well in 82% of cases in the respondents' departments. The instrument was most suitable in the intraoperative phase (82%), followed by the postoperative phase (73%), and was least suitable in the preoperative phase (55%). Nearly all the respondents (92%) indicated that the instrument covered the different dimensions of perioperative nursing relatively well or well. Slightly fewer respondents, 84%, agreed that the instrument succeeded to separate the different dimensions of perioperative nursing relatively well or well.

The concreteness, usability, understandability, clarity, and objectivity of the instrument were also rated by the nurses. The concreteness received the highest CVI value (0.77), while the objectivity received the lowest CVI value (0.74). The rest of the qualities and CVI values were as follows: understandability, 0.76; usability, 0.75; and clarity, 0.75.

## 5. Discussion

### 5.1. Discussion of the Results

The validity of the NI instrument tested in this study received further support and the results are promising, and the results from reliability testing must be interpreted more cautiously.

First, the NI points correlated with the ASA categories given by physicians, but only in the ASA categories of III and IV. Although the NI results did not correlate with low ASA categories (I-II), this does not mean that patients with low ASA categories might not need demanding nursing care. The patients with high ASA categories had high NI requirements during intraoperative and postoperative phase of care. However, for ASA category I or II, patients might still have demanding needs for nursing care. This is because patients' NI points for ASA category I ranged from 6 to 20 both in intraoperative and in postoperative phase. Patients' NI points for ASA category II range intraoperatively from 6 to 22 and postoperatively from 6 to 15. In other words, it should not be taken for granted that patients with low ASA category automatically have less care needs in perioperative settings. This means that we cannot rely on ASA measures alone to evaluate the need for nursing care in the perioperative nursing process.

The surgical procedure may be regarded as simple but patient may have some individual care needs which will make him or her more depending on nursing care. These care needs may be related to one or several categories of our NI instrument. On the other hand, the surgical procedure may be so complex that care needs increase or something unplanned may occur during the procedure. These will influence the total amount of NI points the patient will receive.

The data was scanter in preoperative phase because the preoperative phase did not actualize in every department involved in the study. The patients were taken straight to the operating room from the wards without any caring in the holding area. The results concerning the preoperative phase must therefore be interpreted with caution.

Second, the results concerning the NI instrument's predictive validity are promising. The length of stay in PACU may be predicted with intraoperative NI points and patient's need for intensive care after surgery may be predicted by both intra- and postoperative NI points.

A patient with high intraoperative NI required an extended PACU time and high intraoperative NI also predicted the patient's need for intensive care after the surgical procedure. This result is promising and provides support for the use of NI points in perioperative settings. The instrument's potential to predict the need for lengthened PACU time and the need for ICU care is an important aspect for those who are responsible for nurse staffing. A combination of ASA Physical Status Classification and NI evaluation would provide a more valid estimate of patient's care needs than ASA classification alone.

In addition to the aforementioned predictive validity of the NI instrument, the intraoperative NI correlated positively both with the duration of the surgical procedure and with the time spent in the operating room. Thus, it can be envisaged that by using the classification system systematically and reliably we could create big data that would help us to develop predictive models that could be used for future resource and performance planning.

Third, in each perioperative phase, the agreement percentage of nurses' parallel classifications was over the chosen limit value of 70%. This finding indicates the high interrater reliability of the instrument in general. In the future, more testing with sufficient data is needed to test a consensus between the evaluations at a single department.

According to the nurses who responded to the survey, the instrument tested in this study was comprehensive and sensitive. The qualities of the instrument, including concreteness, usability, understandability, clarity, and objectivity, were considered rather homogenous. This is a suitable result for a totally new instrument with a rather limited usage time of only two months in this case.

However, conclusions must be drawn with care because of the low response rate. It was only 31%, despite the reminder-message we sent. Also, it needs to be kept in mind that, in the preoperative phase, the results may be poorer than in intra- and postoperative phase due to the limited prevalence of patients in the preoperative phase in the different departments. This may have led in difficulties in instrument evaluation.

## 6. Strengths and Limitations of the Study

The data were gathered from nine operating departments from five university hospitals representing different kinds of surgical fields. Thus, the scope was quite wide, confirming the sufficient diversity of our testing environment. Different care contexts are both a strength and a weakness, the latter because in reliability testing and feasibility testing we were forced to handle the data from different departments as a whole. Thus, any conclusions about department level or correlations between the departments cannot be made.

Strength of the study was that the educator of the training sessions had strong clinical background in the field of the study. The content of training sessions was similar in each organization and the training was given by the principal researcher. The patient cases were tailored for each department and were not standardized. This decision was made to help nurses to use the instrument but at the same time it may have affected the consistency of the training.

The NI data were gathered only from patients whose intraoperative phase of care lasted more than 30 minutes. This criterion may have created some bias in the data and, consequently, in our results and conclusions. Also, the study period was limited to two months per each department for practical reasons and may have been too short to capture the diversity of the performance in departments involved. However, the study periods were scheduled together with the participating departments, and they were to cover as normal performance as possible outside the holiday season.

The response rate of only 31% in the feasibility survey may also be considered as a weakness.

## 7. Recommendations for Clinical Practice and Education and Further Research

Patient classification systems will support nurse managers in knowledge-based decision-making relating to staff allocation. At the same time, the use of patient classification system will serve the principles of shared governance by giving to the frontline nurses the possibility of participating in decision-making that relates to their clinical practice.

In nursing education curricula, it must be ensured that the graduated nurses have competence not only in clinical practice but also in describing their practice in a measurable way.

The instrument for the assessment of NI was used in this study only for a limited time period. Because the instrument is designed for daily routine use, a follow-up study could be performed. Due to its validity, reliability, and feasibility, the use of this instrument will likely increase in the near future. As a result, its use will produce more data concerning patients' NI in different perioperative settings and in different phases of perioperative continuum, enabling more detailed testing.

## 8. Conclusions

The instrument tested in this study could be suitable for the assessment of NI based on patients' care needs in perioperative settings. The instrument showed sufficient validity, and the interrater reliability was satisfactory. From the user's point of view, the instrument was regarded as fairly feasible to cover the patient's care needs and also to separate these needs from each other. Different qualities of the instrument were considered adequate and homogenous.

The instrument has the potential to produce information to influence staff allocation and workforce planning. Tentatively, we argue that taking into account the surgical patients' NI will add value to the management of the nursing workforce and the allocation of nurses according to patient care needs to ensure safe and high-quality nursing care. The implementation and ongoing measurement of NI in perioperative settings are essential for the further validation of the instrument.

In conclusion, our findings of the evaluation of surgical patients' NI at different phases of a surgical procedure may improve upon the results obtained based on the patient's ASA category alone.

## Figures and Tables

**Table 1:** ASA Physical Status Classification System according to the American Society of Anaesthesiologists.

Category	Definition
ASA I	A normal healthy patient
ASA II	A patient with mild systemic disease
ASA III	A patient with severe systemic disease
ASA IV	A patient with severe systemic disease that is a constant threat to life
ASA V	A moribund patient who is not expected to survive without the operation
ASA VI	A declared brain-dead patient whose organs are being removed for donor purposes

**Table 2:** Nursing intensity scores in different ASA categories.

Phase	ASA I (*n* = 327) Mean/median (IQR) Min–max	ASA II (*n* = 273) Mean/median (IQR) Min–max	ASA III (*n* = 171) Mean/median (IQR) Min–max	ASA IV (*n* = 85) Mean/median (IQR) Min–max	*p* value^*∗*^
Pre phase	6.8/6.0	7.2/7.0	8.2/8.0	12.3/12.5	<0.0001
	(6.0–7.0)	(6.0–8.0)	(6.0–10.0)	(12.0–13.0)	
	6–11	6–12	6–13	8–16	
	*n* = 153	*n* = 81	*n* = 17	*n* = 6	

Intra phase	9.9/10.0	10.4/10.0	12.1/12.0	16.1/17.0	<0.0001
	(8.0–11.0)	(8.0–12.0)	(9.0–15.0)	(14.0–19.0)	
	6–20	6–22	6–22	7–24	
	*n* = 317	*n* = 267	*n* = 166	*n* = 78	

Post phase	8.4/8.0	8.5/8.0	9.6/9.0	11.1/10.0	<0.0001
	(7.0–10.0)	(7.0–10.0)	(7.0–12.0)	(9.0–14.0)	
	6–20	6–15	6–18	6–19	
	*n* = 268	*n* = 200	*n* = 104	*n* = 21	

IQR = interquartile range; ^*∗*^Kruskal-Wallis test; significant differences in pairwise comparisons between ASA categories with Mann–Whitney *U* tests using Bonferroni-corrected *p* values: In pre phase I versus III (*p* = 0.023), I versus IV (*p* < 0.0001), II versus IV (*p* = 0.0006), and III versus IV (*p* = 0.034); in intra phase I versus III (*p* < 0.0001), I versus IV (*p* < 0.0001), II versus III (*p* < 0.0001), II versus IV (*p* < 0.0001), andn III versus IV (*p* < 0.0001); in post phase I versus III (*p* < 0.0001), I versus IV (*p* = 0.002), II versus III (*p* = 0.004), and II versus IV (*p* = 0.005). Three patients in ASA category V were excluded from the analysis. ASA category VI did not appear in our data.

**Table 3:** Nursing intensity scores in different follow-up units.

Phase	Follow-up unit			*p* value
	Home (*n* = 327) Mean/median (IQR)	Ward (*n* = 273) Mean/median (IQR)	ICU (*n* = 171) Mean/median (IQR)	
Pre phase	7.0/7.0	8.2/8.0	9.0/9.0	0.0006^*∗*^
	(6.0–8.0)	(6.0–9.0)	(8.0–10.0)	
	*n* = 222	*n* = 37	*n* = 2	

Intra phase	9.5/9.0	10.7/10.0	17.1/17.0	<0.0001^#^
	(8.0–11.0)	(8.0–13.0)	(15.0–19.0)	
	*n* = 223	*n* = 511	*n* = 75	

Post phase	8.2/8.0	8.9/8.0	9.5/9.5	0.016^*∗*^
	(7.0–9.0)	(7.0–10.0)	(9.0–10.0)	
	*n* = 220	*n* = 368	*n* = 2	

IQR = interquartile range; ICU = intensive care unit; ^*∗*^Mann–Whitney *U* test. Two patients who were transferred to the ICU were excluded from the analysis. ^#^Kruskal-Wallis test; significant differences in pairwise comparisons between follow-up units with Mann–Whitney *U* tests using Bonferroni-corrected *p* values: Home versus ward (*p* < 0.0001), home versus ICU (*p* < 0.0001), and ward versus ICU (*p* < 0.0001).
